# Association of SNP–SNP Interactions of Surfactant Protein Genes with Pediatric Acute Respiratory Failure

**DOI:** 10.3390/jcm9041183

**Published:** 2020-04-20

**Authors:** Chintan K. Gandhi, Chixiang Chen, Rongling Wu, Lili Yang, Nithyananda Thorenoor, Neal J. Thomas, Susan L. DiAngelo, Debbie Spear, Garrett Keim, Nadir Yehya, Joanna Floros

**Affiliations:** 1 Center for Host defense, Inflammation, and Lung Disease (CHILD) Research, Department of Pediatrics, Pennsylvania State University College of Medicine, Hershey, PA 17033, USA; cgandhi@pennstatehealth.psu.edu (C.K.G.);; 2 Department of Public Health Science, Pennsylvania State University College of Medicine, Hershey, PA 17033, USA; 3 School of First Clinical Medicine, Nanjing University of Chinese Medicine, Nanjing 210023, China; 4 Department of Pediatrics, University of Pennsylvania Perelman School of Medicine, Philadelphia, PA 19104, USA; 5 Department of Obstetrics & Gynecology, Pennsylvania State University College of Medicine, Hershey, PA 17033, USA

**Keywords:** SNP–SNP interaction, Pediatric acute respiratory failure, Surfactant protein gene A1 (*SFTPA1*), *SFTPA2*, *SFTPB*, *SFTPC*, *SFTPD*

## Abstract

The hallmarks of pediatric acute respiratory failure (ARF) are dysregulated inflammation and surfactant dysfunction. The objective is to study association of surfactant protein (SP) genes’ single nucleotide polymorphisms (SNPs) with ARF and its morbidity: pulmonary dysfunction at discharge (PDAD), employing a single-, two-, and three-SNP interaction model. We enrolled 468 newborn controls and 248 children aged ≤ 24 months with ARF; 86 developed PDAD. Using quantitative genetic principles, we tested the association of SP genes SNPs with ARF and PDAD. We observed a dominant effect of rs4715 of the *SFTPC* on ARF risk. In a three-SNP model, we found (a) 34 significant interactions among SNPs of *SFTPA1, SFTPA2,* and *SFTPC* associated with ARF (*p* = 0.000000002–0.05); 15 and 19 of those interactions were associated with increased and decreased risk for ARF, respectively; (b) intergenic SNP–SNP interactions of both hydrophobic and hydrophilic SP genes associated with PDAD (*p* = 0.00002–0.03). The majority of intra- and intergenic interactions associated with ARF involve the *SFTPA2* SNPs, whereas most of the intra- and intergenic interactions associated with PDAD are of *SFTPA1* SNPs. We also observed a dominant effect of haplotypes GG of *SFTPA1* associated with increased and AA of *SFTPC* associated with decreased ARF risk (*p* = 0.02). To the best of our knowledge, this is the first study showing an association of complex interactions of SP genes with ARF and PDAD. Our data indicate that SP genes polymorphisms may contribute to ARF pathogenesis and subsequent PDAD and/or may serve as markers for disease susceptibility in healthy children.

## 1. Introduction

Acute respiratory failure (ARF), defined as the failure of the respiratory system to provide oxygen and/or eliminate carbon dioxide, is a common cause of critical illness in children [1]. ARF can also be defined based on blood gas abnormalities: partial pressure of carbon dioxide > 55 mm Hg, partial pressure of oxygen < 60 mm Hg, and/or pH < 7.35 [2]. ARF is more common in infants and young children compared to adults due to anatomical and physiological disadvantages [3]. The incidence of ARF is inversely related to age and it is one of the common cause of death in children ≤ 24 months [4]. Without prompt intervention, ARF is associated with significant morbidity and mortality [2]. Single center cohort studies have shown that many children among the ARF survivors develop pulmonary dysfunction at discharge (PDAD) [5,6]. Respiratory infections and direct lung injury are the most common causes of ARF in children [4]. The pathophysiology of ARF is multifactorial and depends on underlying etiology, co-morbidities, and the host’s response. The hallmarks of respiratory causes of ARF are dysregulated inflammation and concurrent surfactant dysfunction [2]. Moreover, there is a considerable heterogeneity in the progression and severity of the disease. Therefore, it is conceivable that an underlying genetic predisposition, along with environmental factors, may contribute to the cause and pathogenesis of complex diseases such as pediatric ARF and its progression. Nonetheless, the role of genetics in pediatric ARF is under studied.

Pulmonary surfactant is a lipoprotein complex composed of 90% lipids and 10% surfactant proteins (SPs). Surfactant protein (SP)-A, SP-B, SP-C, and SP-D compose the protein portion of pulmonary surfactant. The hydrophobic surfactant proteins (SP-B and -C) play an important role in reducing the surface tension and SP-B is essential for life [7]. In contrast, the hydrophilic SPs (SP-A and -D) are important for innate immunity and host defense against infections and air pollutants [8,9,10], although SP-A also plays a role in surfactant related functions [11]. In humans, unlike in rodents, there are two similar genes, *SFTPA1* and *SFTPA2,* located on chromosome 10 encoding SP-A1 and SP-A2, respectively. Several polymorphisms have been described in the coding region of both the *SFTPA1* and *SFTPA2* genes [12]. SP-B, SP-C, and SP-D are each encoded by a single gene and several polymorphisms have been described for each [13,14].

Single nucleotide polymorphisms (SNPs) of the SP genes have been identified and shown to associate with various acute and chronic pulmonary diseases, such as neonatal respiratory distress syndrome (RDS) [15,16,17,18], cystic fibrosis (CF) [19], chronic obstructive pulmonary disease (COPD) [20], and acute respiratory distress syndrome (ARDS) [21]. Furthermore, SP gene polymorphisms are shown to associate with pulmonary diseases of infectious etiology such as tuberculosis [22] and respiratory syncytial virus (RSV) [23]. The most common cause of ARF in children is infection, particularly pneumonia and bronchiolitis [24]. Surfactant dysfunction and inactivation have been observed in pediatric ARF [25,26]. Moreover, in adult ARDS, the SP-B level is reduced in bronchoalveolar lavage, and its ability to reduce the surface tension is also decreased [27]. Therefore, it makes surfactant proteins the perfect candidates to study the role of genetics in pediatric ARF, since collectively they contribute to innate host defense and surfactant function; both of these can be dysregulated and dysfunctional in ARF.

In multifactorial diseases such as pediatric ARF, complex interactions of genetic and environmental factors are implicated in the pathogenesis of the diseases. It is also known that a given genetic variant in the presence of another genetic variant can either enhance or nullify its effect [28,29,30,31]. We selected 14 well-characterized SNPs of *SFTPA1, SFTPA2, SFTPB, SFTPC,* and *SFTPD* genes in order to investigate associations of these polymorphisms (a) with pediatric ARF, and (b) with disease severity and progression based on the development of PDAD. We hypothesized that natural genetic variants of SPs are associated with ARF and its morbidity.

## 2. Subjects and Methods

### 2.1. Study Population

Cases: We prospectively enrolled 250 previously healthy children aged 0–24 months admitted in the pediatric intensive care units (PICUs) of 10 children’s hospitals with a primary diagnosis of ARF over five consecutive years. Inclusion criteria are ARF due to respiratory cause with the presence of at least one of the following three criteria: (1) clinical findings consistent with lower respiratory tract illness, (2) focal or diffuse infiltrative pulmonary process on chest radiograph, or (3) radiographic evidence of air trapping. The cause of ARF was determined at the discretion of the site investigators. Children with aspiration or primary upper airway disease as the etiology of ARF, or with pre-existing neurologic, neuromuscular or cardiac disease were excluded. Since we were interested in previously healthy children, we excluded children with a history of (1) premature birth <35 weeks of gestation, (2) chronic oxygen and/or mechanical ventilation (MV) dependency, (3) bronchodilators, inhaled and/or systemic corticosteroids use prior to present illness, (4) previous PICU admission and/or mechanical ventilation course other than for surgical procedure, and (5) receipt of exogenous surfactant. Two children subsequently were diagnosed with CF, and therefore excluded from the analysis, yielding the final cohort of 248 children. All demographic and clinical data were collected prospectively for children with ARF. Positive tracheal aspirate, urine, and/or blood culture was considered as positive bacterial culture.

Children with ARF were further stratified based on the development of PDAD, defined a priori as a need of at least one of the following at 28 days of admission or hospital discharge, whichever comes first: MV, supplemental oxygen, bronchodilators or steroids (inhaled and/or systemic).

Controls: 468 unrelated term newborns delivered at Penn State Hershey Medical Center served as a control.

We collected blood samples of study participants after obtaining informed consent from a parent or legal guardian. This study was approved by the institutional review board of the Human Subject Protection Office of the Pennsylvania State University College of Medicine and participating sites.

### 2.2. DNA Isolation

We extracted genomic DNAs from blood samples using QIAamp Blood kit (Qiagen, Valencia, CA USA) following the manufacturer’s instructions, as described previously [12].

### 2.3. Genetic Variations Selection for this Study

We selected a total of 14 target SNPs of surfactant protein genes *SFTPA1*, *SFTPA2*, *SFTPB*, *SFTPC*, and *SFTPD*, all previously shown to be associated with various lung diseases. The 14 SNPs include five SNPs from *SFTPA1*: rs1059047, rs1136450, rs1136451, rs1059057, and rs4253527; four SNPs from *SFTPA2*: rs1059046, rs17886395, rs1965707, and 1965708; one SNP from *SFTPB*: rs1130866; two SNPs from *SFTPC*: rs4715 and rs1124; and two SNPs from *SFTPD*: rs721917 and rs2243639.

### 2.4. Genotype Analysis

We used polymerase chain reaction-restriction fragment length polymorphism (PCR-RFLP) to analyze the *SFTPA1*, *SFTPA2*, *SFTPD* [12,21], *SFTPB* [21,32], and *SFTPC* [33] gene polymorphisms, as described earlier [12]. The PCR primer sequences, restriction enzymes and the method is described here in detail [19]. As samples were received from participating sites, each was given a sequential laboratory number with no other identifiers. All samples were genotyped together in a blinded fashion with those performing analysis unaware of the clinical status. We therefore believe that there was no bias in genotyping.

### 2.5. Statistical Analysis

We used an SNP–SNP interaction method in a case-control setting by integrating quantitative genetic principles to study associations of SP genes polymorphisms with pediatric ARF and its morbidity [34]. Since this novel SNP–SNP interaction method could help understand the contribution of each gene to disease, we used this statistical method in a single-, two-, and a three-SNP model for the present study. We used the Wang et al.’s [34] approach (provides computer code (written in R) for public use), which is a more efficient approach compared to more traditional methods [35] to test and estimate the genetic effects of the 14 SNPs. This approach integrates the principle of quantitative genetics, enabling the decomposition of the overall genetic effect into different components: the additive (a) and dominant genetic effect (d) of each SNP, additive × additive (a × a), additive × dominant (a × d), dominant × additive (d × a), and dominant × dominant epistatic effects (d × d) between the two SNPs. For any three SNPs, we tested for the following eight 3 SNP–SNP epistasis effects: the additive × additive × additive (a × a × a), additive × additive × dominant (a × a × d), additive × dominant × additive (a × d × a), dominant × additive × additive (d × a × a), additive × dominant × dominant (a × d × d), dominant × additive × dominant (d × a × d), dominant × dominant × additive (d × d × a), and dominant × dominant × dominant (d × d × d). We tested all the possible two- and three- SNP–SNP interactions for this study and reported significant interactions with *p* < 0.05. To account for multiple testing, the false discovery rate (FDR) is controlled at 5%, using the Benjamini–Hochberg method [36,37]. The Cochran’s and Mantel–Haenszel test was used to adjust clinical variables and calculate odds ratios (OR) with 95% of confidence interval (95% CI) [38]. By estimating the role of each of these components, this approach can provide a better understanding of the inheritance mode by which SNPs impact the disease. To analyze SNP–SNP interactions, we calculated *p*-values for each genetic component and adjusted for FDR. We also used the Lin et al.’s method to study associations of haplotypes with ARF compared to newborn controls in a two-SNP haplotype estimation model [39].

This study includes 250 cases and 468 controls, which are converted into a 2 × 2 contingency table where various type of epistatic interactions at different orders are tested. According to our simulation studies [34], a 100 × 100 sample size combination in an epistatic case-control study can reach a power of >0.80 to detect significant associations from a 2 × 2 setting. Therefore, our current sample size combination should provide an adequate power to detect all possible significant epistatic interactions.

## 3. Results 

### 3.1. Clinical Characteristics of ARF Study Group

**Figure 1:**
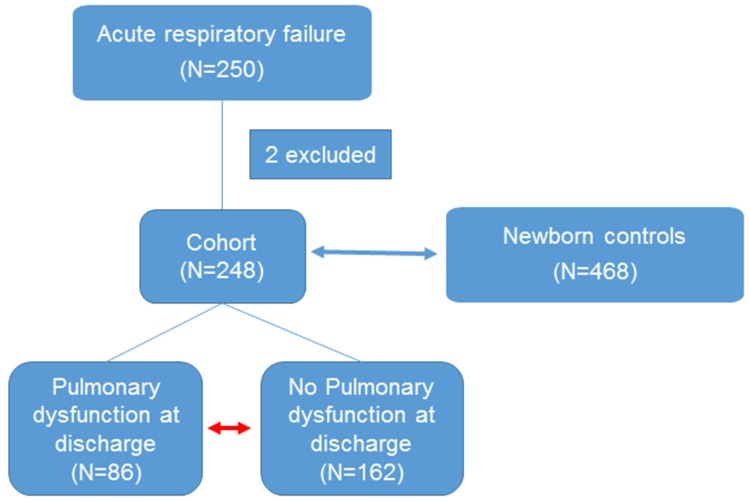
Study outline. Previously healthy children with acute respiratory failure (ARF) are enrolled in the study (*n* = 250). Two children subsequently diagnosed with cystic fibrosis were excluded from final analysis (*n* = 248). The statistical analysis (shown by a blue double headed arrow) is done between the cases (ARF, *n* = 248) and controls (healthy newborns, *n* = 468). Then, further analysis was performed between the cohort of children with and without pulmonary dysfunction at discharge (PDAD) shown in red double headed arrow (*n* = 86 vs 162).

Figure 1 shows the study outline. Table S1 shows the baseline characteristics of the ARF patients (*n* = 248) and newborn control (*n* = 468). In summary, there are fewer females (*n* = 99/248, 39%) and non-Hispanic (*n* = 196/248, 79%) children in the ARF cohort compared to newborn controls (female = 240/468, 51% and non-Hispanic = 459/468, 98%), *p* = 0.000. There is not a statistically significant difference in race between the groups.

Table 1 shows the baseline characteristics of those who did/did not develop PDAD. Out of 248 ARF children, 86 developed PDAD: four required MV, 16 supplemental oxygen, 36 oral/inhaled steroids, and 66 bronchodilators. Those who developed PDAD were older, more likely to have positive bacterial culture, had higher nadir oxygen index, required higher ventilator support, and longer duration of support (ventilator, oxygen, and PICU days).

### 3.2. SNP–SNP Interaction

The SNPs studied here are not uncommon variants [12]. Table 2 and Tables S2–S4 depict the significant associations after FDR correction for multiple comparisons. The column “interaction” in supplementary Tables represents the SNP–SNP interactions that could be intragenic, i.e., between the SNPs of an individual gene (shown with superscript letter “a”), or intergenic, i.e., between the SNPs of different genes. The letter “a” is for additive and “d” is for dominant effect of that particular SNP. The number following “a” or “d” indicates the position of the corresponding SNP, e.g., a1 × d2 × d3 shows the additive effect of SNP1 and dominant effects of SNPs two and three. Though number of dominant effects have not shown to associate with a biological impact, we sorted the three-loci epistasis into four groups interactions for simplicity and ease of understanding out of eight possible forms: (1) no dominant effect-a1 × a2 × a3; (2) one dominant effect-a1 × a2 × d3, a1 × d2 × a3, d1 × a2 × a3; (3) two dominant effects-a1 × d2 X d3, d1 × a2 × d3, d1 × d2 × a3; and (4) three dominant effects-d1 × d2 × d3.

### 3.3. Association of SP Genes SNPs with ARF Compared to Newborn Controls after Adjusting for Sex and Ethnicity

We used single-, two-, and three-SNP model to study association of SP gene SNPs with ARF compared to newborn controls and adjusted for sex and ethnicity due to a significant difference between the groups. In a single-SNP model, rs4715 (C/A) of *SFTPC* had a dominant effect on ARF risk (*p* = 0.03, OR (CI) – 2.4 (1.3–4.3)). A dominant effect means that the heterozygote (CA) at this SNP increases the risk of ARF beyond the mean of two homozygotes (CC, AA). In a two-SNP model, we did not observe any significant SNP–SNP interaction associated with ARF. In a three-SNP model, we found 34 significant interactions among SNPs of *SFTPA1, SFTPA2,* and *SFTPC* SNPs associated with ARF (*p* = 0.000000002–0.05), shown in Table 2. We did not observe any significant interactions of *SFTPB* and *SFTPD* SNPs associated with ARF. Of the 34 interactions, none showed a significant interaction with only additive effects (a1 × a2 × a3) in a three-SNP model. All the significant three-loci epistasis had at least one SNP with a dominant effect. Out of the 34 total interactions, 15 are associated with increased risk and 19 are associated with decreased risk of ARF.

For the 15 interactions with increased ARF risk, we observed (a) that all but one interaction involved SNPs of the *SFTPA1* and *SFTPA2* genes only, and that single interaction included the rs4715 of the *SFTPC* that had a dominant effect and SNPs of *SFTPA1* and *SFTPA2*; (b) four intragenic interactions of *SFTPA2* SNPs; and (c) out of those 15 interactions, we identified (i) four interactions with one dominant effect of SNPs (rs1965707, rs1965708) of *SFTPA2* and an additive effect of SNPs of *SFTPA1* (*p* = 0.007–0.03, OR = 2.2–2.5, CI = 1.3–4.7), (ii) 10 interactions with two dominant effects (*p* = 0.007–0.04, OR = 1.6–4), and (iii) one interaction with three dominant effects of *SFTPA1* and *SFTPA2* (*p* = 0.00005, OR = 1.6).

For the 19 interactions that are associated with lower risk for ARF, we observed (a) fourteen intergenic interactions among SNPs of *SFTPA1, SFTPA2,* and *SFTPC*; (b) five intragenic interactions of SNPs of the *SFTPA1* (*n* = 2) and *SFTPA2* (*n* = 3); (c) out of the 19 interactions, we identified (i) one intragenic interaction of *SFTPA2* with one dominant effect (rs1059046 × rs17886395 × rs1965708, a1 × a2 × d3, *p* = 0.03, OR = 0.4), (ii) four significant interactions with two dominant effects (*p* = 0.008–0.05, OR = 0.2–0.6), and (iii) fourteen interactions with three dominant effects (*p* = 0.000000002–0.05, OR = 0.2–0.9).

Considering the percentage (almost 70%) of *SFTPA2* SNPs out of the total SNPs involved in intragenic and intergenic interactions with increased ARF risk, we speculate that innate immunity functions imparted by *SFTPA2* gene variants under certain conditions contribute to the pathogenesis of ARF.

### 3.4. Haplotype Association with ARF Risk

**Figure 2:**
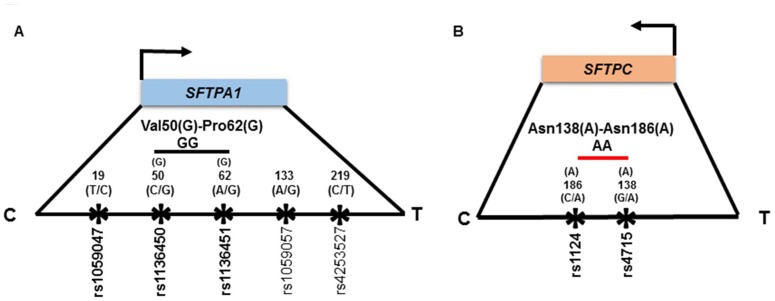
(**A**) shows schematic presentation of *SFTPA1* and (**B**) shows *SFTPC* gene and transmission of haplotypes in ARF. The relative location of genes is shown from centromere (**C**) to telomere (T). In each panel, the number above the solid black line indicates the amino acid number and corresponding nucleotide change shown in parenthesis. The SNP id is shown below the black line. The arrows indicate transcriptional orientation. The transmitted haplotypes and corresponding amino acid changes are shown in bold in a two-SNP model. A shows the location of the *SFTPA1* gene in blue on the long arm of chromosome 10. Haplotype “GG” shown in black line, constituted by rs1136450 Val(G) at codon 50 and rs1136451 Pro(G) at codon 62 within the *SFTPA1* gene, are associated with increased ARF risk in a previously healthy children, *p* = 0.02, OR = 9.3 (1.7–49.6). B shows the *SFTPC* gene in orange and its orientation in terms of the centromere and telomere. The haplotype “AA”, shown in red line, constituted by rs4715 Asn(A) at codon 138 and rs1124 Asn(A) at codon 186 within the *SFTPC* gene, is shown to associate with lower risk for ARF, *p* = 0.02, OR = 0.4 (0.2–0.7). Both significant haplotypes have a dominant effect on ARF. Of note, the physical location of the transmitted SNPs in these haplotypes is very close, as shown in Figure.

Ne×t, we wanted to study the haplotype transmission that is associated with ARF risk using Lin et al.’s haplotype estimation method in a two-SNP model [39]. Figure 2 shows the haplotypes that are estimated to get transmitted from parents to children with ARF. For example, the rs1136450 (C/G) of *SFTPA1* at codon 50 (Leu/Val) and the rs1136451 (A/G) of *SFTPA1* at codon 62 (Pro/Pro, no change in amino acid) can be transmitted from a single parent in four forms: CA, CG, GA, and GG. These haplotypes can have an additive, recessive, or dominant effect, depending on the OR > or < 1 that indicates increased or decreased risk, respectively, for the disease. In this example, we found that the haplotype “GG” acts as the risk haplotype, exerting a dominant effect on ARF risk, *p* = 0.02, OR = 9.3 (1.7–49.6). That is, the combination of GG displays a higher ARF risk compared to the combination of non-risk haplotypes (CA, CG, and GA). In contrast, the haplotype “AA” from Asn(A) at codon 138 (Thr/Asn) and Asn(A) at codon 186 (Ser/Asn) within the *SFTPC* gene have a dominant effect and are associated with lower risk for ARF, *p* = 0.02, OR = 0.4 (0.2–0.7).

### 3.5. Association of SP Genes with the PDAD Subgroup Compared to no PDAD after Individual Adjustments of Variables

To gain further insight into the contribution of SP genes to the progression of the disease, we divided the cohort into subgroups: (a) those who develop PDAD (*n* = 86), and (b) those who did not develop PDAD (*n* = 162) (see method section). Age, positive bacterial culture, and ventilator days were significantly different between the subgroups, therefore, these were adjusted separately for subgroup analysis (Table 1).

#### 3.5.1. Age

Children who did not develop PDAD were younger than those who developed PDAD (*p* = 0.001). We observed one intergenic interaction with two dominant effects that increased the risk of PDAD, *p* = 0.04, OR = 3.1 (1.7–5.9), shown in Table S2. Out of the 37 intergenic interactions with three dominant effects, we observed nine interactions associated with increased risk of PDAD (*p* = 0.00005 – 0.02, OR = 1.2–3.6) and 28 interactions associated with lower risk for PDAD (*p*= 0.00007–0.04, OR = 0.3–0.9). No intragenic interactions were observed (Table S3). Out of eight interactions among the hydrophilic SPs alone, three and five interactions were associated with increased and decreased risk of PDAD, respectively, among ARF survivors. Seven interactions among hydrophilic SPs were between SNPs of either *SFTPA1* and/or *SFTPA2*, and *SFTPD,* and one between SNPs of *SFTPA2* and *SFTPA1* (rs1059046 × rs1965707 × rs1136540, *p* = 0.03, OR = 0.6 (0.5–0.9)). In both the two-dominant and the three-dominant effect, all the interactions except one involved at least one SNP of either *SFTPA1* or *SFTPA2*. One interaction without *SFTPA1* or *SFTPA2* had two SNPs of *SFTPC* and one of *SFTPD* (rs721917), *p* = 0.03, OR = 0.6 (0.3–0.9).

#### 3.5.2. Positive Bacterial Culture

Out of 248 children with ARF, 195 children had either blood, urine and/or tracheal culture available (Table 1). Among the PDAD subgroup, 82% had positive bacterial culture compared to 63% of those who did not develop PDAD, *p* = 0.009.

Table S3 shows significant SNP–SNP interactions of SP genes with PDAD after adjusting for positive bacterial culture. We found a total of 13 interactions with two dominant effects (*p* = 0.0002–0.04). All interactions are intergenic and between the SNPs of both hydrophobic and hydrophilic SPs. Out of 13 interactions, 11 were associated with increased risk for PDAD, *p*= 0.0002–0.04, OR = 2.5–4 (1.5–9.5), whereas two interactions with lower risk for PDAD, *p* = 0.0004–0.002, OR = 0.2 (0.1–0.5). Furthermore, all but two interactions involved at least one SNP of *SFTPA1*.

We observed 57 interactions with three dominant effects (*p* = 0.00008–0.05). Out of them, 13 interactions were associated with increased risk for PDAD, *p* = 0.0001–0.02, OR = 1.8–2.7 (1.2–4.1), whereas 44 interactions with lower risk for PDAD, *p* = 0.00008–0.05, OR = 0.4–0.6 (0–0.9). rs1059047 and rs1136451 of *SFTPA1* had maximum interactions with SNPs of other SP genes. All but two interactions had a SNP of either the *SFTPA1* or *SFTPA2* gene. The remaining two interactions included both SNPs of *SFTPD* (rs721917, rs2243639) and the *SFTPC* (rs721917 × rs4715 × rs1124, *p* = 0.01; rs2243639 × rs4714 × rs1124, *p* = 0.003), and both were associated with lower risk for PDAD (OR = 0.5–0.6). Similarly, the same two interactions of *SFTPD* and *SFTPC* were associated with lower risk for PDAD after adjusting for ventilator days as well (see below Table S4, *p* = 0.03 and 0.01, respectively, OR = 0.6). We observed two intragenic interactions among SNPs of the *SFTPA1*; however, the interaction (rs1136450 × rs1136451 × rs4253527, *p* = 0.002) was associated with increased risk, OR = 2.5 (1.6–3.9), whereas rs1059047 × rs1136451 × rs4253527, *p* = 0.01, with lower risk for PDAD OR = 0 (0–0.5). Interestingly, the intragenic interaction rs1136450 × rs1136451 × rs4253527 with three dominant effects of *SFTPA1* was associated with increased risk for PDAD after adjusting for ventilator days as well, *p* = 0.04, OR = 1.7 (1.2–2.5) (Table S4).

#### 3.5.3. Ventilator Days

Children who develop PDAD needed longer ventilator support compared to those who did not develop PDAD, *p* = 0.004. Table S4 shows associations of the *SFTP* genes with PDAD after adjusting for ventilator days. We observed 47 interactions with three dominant effects. Out of 47 interactions, 12 were associated with increased risk (*p* = 0.0001, OR = 1.7–2.5) and 35 were associated with decreased risk (*p* = 0.000005–0.05, OR = 0–0.6) for PDAD. Of note, we observed 22 out of 47 significant interactions to be similar between ventilator days and positive bacterial culture adjustment and the majority of these common interactions were associated with lower risk for PDAD (Tables S3 and S4). All interactions were intergenic except one intragenic interaction of *SFTPA1* SNPs as mentioned above. We observed 10 interactions among the SNPs of hydrophilic SPs alone.

### 3.6. Common SNP–SNP Interactions

#### 3.6.1. Association with ARF Compared to Controls and PDAD Compared to No PDAD

**Figure 3:**
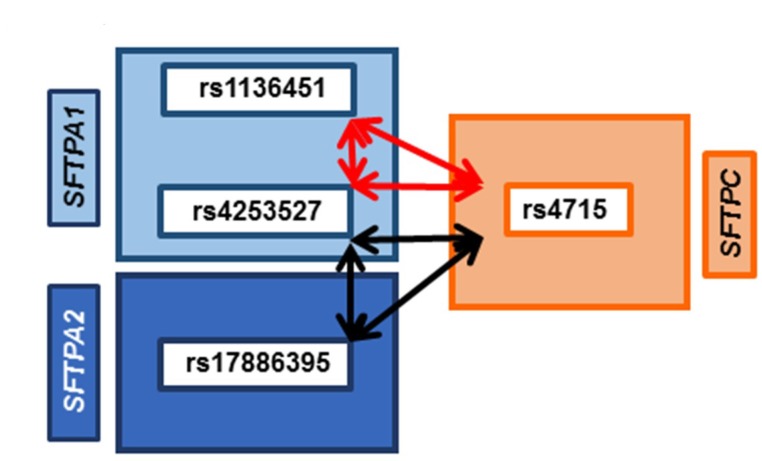
Common association of SNP–SNP interactions with ARF and PDAD. Figure shows SNP–SNP interactions (black and red double head arrows) found in common in two sets of comparisons. In one, comparison between pediatric acute respiratory failure (ARF) to newborn controls; an odds ratio (OR) of 0.5 indicates that these interactions associate with lower risk for ARF. In the other, comparison between those with pulmonary dysfunction at discharge (PDAD) subgroup compared to those who did not develop PDAD after adjusting for age, positive bacterial culture, and number of ventilator days, an OR of 2 to 2.5 indicates that these interactions associate with increased susceptibility of ARF survivors to develop PDAD. The SNP of the surfactant gene *SFTPC*, encoding the hydrophobic SP-C, is depicted within the orange rectangle on the right side; on the left, SNPs of the surfactant genes, *SFTPA1* and *SFTPA2*, encoding the hydrophilic surfactant proteins SP-A1 and SP-A2, are depicted within the light- and dark-blue rectangle, respectively. Arrows represent the significant interactions in a three-SNPs model. These interactions are intergenic and dominant × dominant × dominant (d1 × d2 × d3) among the SNPs of *SFTPA1*, *SFTPA2*, and *SFTPC*. Red arrows represent the d1 × d2 × d3 interactions among two SNPs of *SFTPA1* and one SNP of *SFTPC* (rs1136451 × rs4253527 × rs4715, *p* = 0.00002–0.008), while black arrows represent three SNP interactions among the SNPs of *SFTPA2*, *SFTPA1*, and *SFTPC* (rs17886395 × rs4253527 × rs4715, *p* = 0.0001–0.005). Of note, rs4253527 (*SFTPA1*) and rs4715 (*SFTPC*) are common in both interactions and represent both hydrophilic and hydrophobic surfactant proteins.

Figure 3 shows significant intergenic interactions among SNPs of *SFTPA1*, *SFTPA2*, and *SFTPC*, where each SNP had a dominant effect. Compared to newborn controls (*n* = 468), the OR of 0.5 (CI – 0.3–0.8) indicates that these interactions are associated with lower risk for ARF (*n* = 248), *p* = 0.008–0.01. The same two interactions were significantly associated with PDAD (*n* = 86) among the ARF survivors compared to those who did not develop PDAD (*n* = 162) after adjusting for age, positive bacterial culture, and ventilator days in a three SNP model (*p* = 0.000002–0.01, Tables S2–S4). However, in this case, the OR is 2 to 2.5 (CI – 1.2–3.3), indicating that though these interactions associate with lower risk for ARF, in this group comparison, they associate with increased risk for the progression of disease (PDAD). Of note, rs4253527 (*SFTPA1*) and rs4715 (*SFTPC*) are in common in both of these interactions, and interact with rs1136451 (*SFTPA1*) or rs17886395 (*SFTPA2*), respectively. These common intergenic interactions involve SNPs of both hydrophobic and hydrophilic SPs.

#### 3.6.2. Associations with PDAD Compared to No PDAD after Adjusting for Age, Positive Bacterial Culture, and Ventilator Days

**Figure 4:**
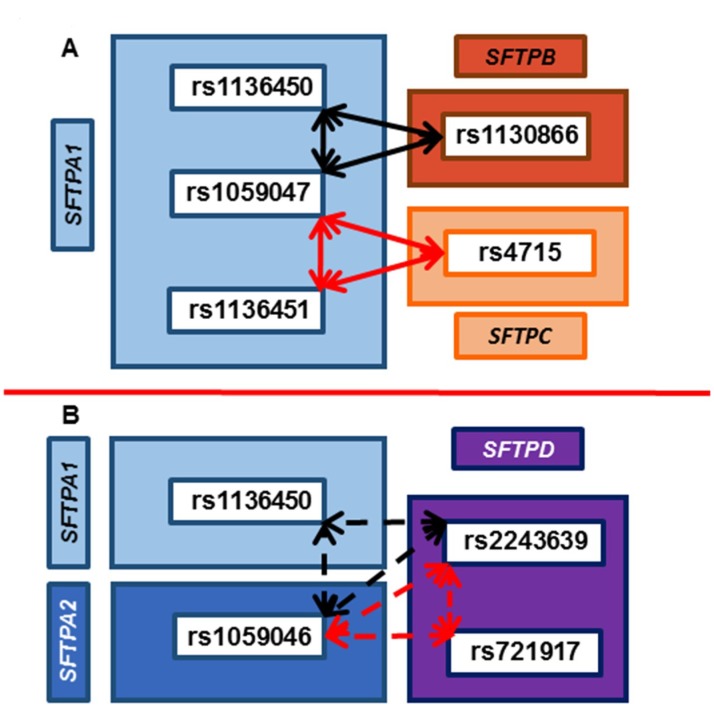
Association of SNP–SNP interactions of SP genes with PDAD. (**A**) shows interaction of SNPs of *SFTPA1*, *SFTPB*, and *SFTPC* genes and (**B**) shows interaction of SNPs of hydrophilic SPs (*SFTPA1, SFTPA2*, and *SFTPD*) alone. SP gene SNP–SNP interactions associated with PDAD compared to those who did not develop PDAD after adjusting for age, positive bacterial culture, and ventilator days. All interactions are intergenic and in a dominant form. In A, on the right, one SNP of the surfactant gene *SFTPB* and one SNP of the *SFTPC* gene, encoding the hydrophobic SP-B and SP-C, respectively, is shown within the dark and light orange rectangle, respectively. The *SFTPA1* gene is shown in light blue on the left side. Solid arrows represent interactions between the hydrophilic *SFTPA1* gene, and the hydrophobic *SFTPB* and *SFTPC* genes (d1 × d2 × d3 = rs1059047 × rs1136450 × rs1130866, *p* = 0.003–0.02, rs1059047 × rs1136451 × rs4715, *p* = 0.003–0.03). The odds ratio (OR) of less than one (0.4–0.6) indicates that both of these interactions associate with lower risk for PDAD. In B, the *SFTPD* gene is shown in purple on the right side. *SFTPA1* and *SFTPA2* genes are shown in light and dark blue, respectively, on the left side. Dotted arrows represent interactions among SNPs of the hydrophilic surfactant proteins, *SFTPA2*, *SFTPA1*, and *SFTPD* (rs1059046 × rs1136450 × rs2243639, *p* = 0.005–0.02, and rs1059046 × rs721917 × rs2243639, *p* = 0.00005–0.01, OR = 2.3). The OR of rs1059046 × rs1136450 × rs2243639 interaction is less than 1 (0.5–0.6), indicating that this interaction associate with lower risk for PDAD. In contrast, the OR of rs1059046 × rs721917 × rs2243639 interaction is more than 1 (1.9–2.3) indicating that this interaction is associated with increased susceptibility for PDAD.

Figure 4 shows four common intergenic interactions associated with PDAD (*n* = 86) compared to no PDAD (*n* = 162) after adjusting for all variables separately (age, positive bacterial culture, and ventilator days). All interactions are with three dominant effects. Figure 4A shows that rs1059047 (*SFTPA1*) interacts with another SNP of *SFTPA1* (rs1136450 or rs1136451) and with SNPs of hydrophobic SPs (rs1130866 of *SFTPB* and rs4715 of *SFTPC*), *p* = 0.003–0.02 and 0.003–0.03, respectively. Both of these interactions associate with lower risk for PDAD, OR = 0.6. In contrast, another two interactions (Fig 4B) among SNPs of hydrophilic SPs (*SFTPA1*, *SFTPA2*, and *SFTPD*) alone, in which rs1059046 of *SFTPA2* and rs2243639 of *SFTPD* are in common, and interact with rs1136450 of *SFTPA1* in one reaction and rs721917 of *SFTPD* in another reaction. The interaction rs1059046 × rs1136450 × rs2243639 associates with lower risk for PDAD, OR = 0.6; whereas, the interaction rs1059046 × rs721917 × rs2243639 associated with higher risk for PDAD, OR = 1.9–2.3.

In summary, we observed an association of surfactant proteins SNPs with ARF and its progression such as PDAD. rs4715 of the *SFTPC* was associated with ARF in a single SNP model. We did not find associations of SP gene SNPs with ARF and PDAD in a two-SNP model. The majority of the interactions associated with increased ARF risk in a three-SNP model are of dominant effects of *SFTPA2* and interact with *SFTPA1*, whereas all interactions that associate with lower risk are among SNPs of the *SFTPA1*, *SFTPA2*, and *SFTPC* genes. We did not observe any association of *SFTPB* and *SFTPD* with ARF in a single-, two-, and three-SNP model. Furthermore, the majority of intragenic interactions associated with increased ARF risk are among SNPs of the *SFTPA2* gene, whereas all intragenic interactions of *SFTPA1* are associated with decreased ARF risk. In contrast, the majority of the interactions associated with increased risk of PDAD are of dominant effects of *SFTPA1* and interact with the *SFTPA2* gene. In general, the majority of significant interactions associated with PDAD involved SNPs of both hydrophilic and hydrophobic SPs. The intragenic interaction of SNPs of the *SFTPA1* are associated with increased risk for PDAD. We did not observe any significant interactions among SNPs of the hydrophobic SPs alone. Since the majority of the interactions associated with ARF involve SNPs of *SFTPA1* and/or *SFTPA2*, we speculate that innate immunity related functions imparted by SP-A1/SP-A2 variants play a critical role in ARF. In contrast, the majority of interactions associated with PDAD involve SNPs of both hydrophilic and hydrophobic SPs and we speculate that the surfactant-related functions imparted by SP-A, in particular SP-A1, SP-B and SP-C variants, further enhance the disease progression to PDAD.

## 4. Discussion

ARF is a multifactorial disease with a complex pathophysiology. Dysregulated inflammation and surfactant dysfunction are central to various pulmonary diseases including pediatric ARF [2]. Therefore, we hypothesized that natural genetic variants of SPs are associated with ARF and its morbidity. Our findings demonstrate association of single SNP, SNP–SNP interactions and haplotypes of SP genes with ARF. Similarly, SNP–SNP interactions were associated with PDAD after adjusting for age, bacterial culture positivity, and ventilator days. We observed association of *SFTPC* with ARF in a single SNP model. We did not observe any association with ARF and PDAD in a two-SNP model. We observed association of SNPs of *SFTPA1, SFTPA2*, and *SFTPC* genes with ARF and all five SP genes with PDAD in a three-SNP model. The majority of SNP interactions associated with increased ARF risk involve *SFTPA2* SNPs, while the majority of SNP interactions associated with PDAD risk involve *SFTPA1*. Specifically, we found that (a) all interactions have at least one dominant effect but most have three dominant effects, (b) almost all intergenic interactions associated with increased ARF risk involve *SFTPA1* and *SFTPA2* genes, whereas, all significant intergenic interactions associated with increased PDAD risk involve both hydrophobic and hydrophilic SP genes, (c) intragenic interactions are among SNPs of the *SFTPA1* and *SFTPA2* genes, (d) *SFTPC* SNPs are involved in more interactions than the *SFTPB* SNPs in PDAD, and (e) The haplotype GG of *SFTPA1* that has a dominant effect on ARF associates with higher risk, and the AA of *SFTPC* has a dominant effect but associates with lower ARF risk. These findings indicate that SP genetic variants are associated with ARF and its morbidity and that certain haplotypes contribute to the susceptibility of ARF. If confirmed, the markers identified in this study may be used to distinguish otherwise healthy children at risk for ARF and subsequent PDAD who could benefit from aggressive therapy at the onset of symptoms.

### 4.1. Association of SFTPC with ARF in a Single-SNP Model

Though not essential for life, the SP-C protein is extremely hydrophobic and along with SP-B plays a role in reducing surface tension. The *SFTPC* gene sequence is highly conserved, but two common polymorphisms, rs4715 at amino acid residue 138 (C/A) and rs1124 at amino acid residue 186 (G/A), have been linked to neonatal RDS in the Finnish premature neonates (<34 weeks), particularly if adjusted for sex [40]. Though these SNPs were associated with RDS, they were not associated with bronchopulmonary dysplasia (progression of RDS) at 36 weeks. Similarly, we observed that rs4715 of *SFTPC* had a dominant effect on ARF risk after adjusting for sex and ethnicity but was not associated with PDAD (progression of ARF) in our study, indicating that this particular SNP is a risk factor for acute pulmonary diseases but not for disease progression. Because very little is known about the functional impact of polymorphisms, we can just speculate about the effects of these polymorphisms on pulmonary diseases such as ARF. Studies of SP-C deficient mice have shown that SP-C is important for stabilization and recruitment of phospholipids in surfactant [41]. Therefore, we can speculate that SP-C polymorphism could decrease the stability of functional surfactant and in turn increase susceptibility of an individual to ARF in response to direct lung injury.

### 4.2. Association of Interactions of SNPs of SP Genes with ARF and PDAD

Although the single- and the two-SNP model did not identify any significant interactions, the three-SNP model did, underscoring the likely complexity of SP gene interactions with ARF and PDAD. Almost all the interactions that were associated with increased risk of ARF were among the SNPs of the *SFTPA1* and *SFTPA2* genes, whereas interactions of these two genes with other genes (*SFTPB, SFTPC*, and *SFTPD*) associated with decreased ARF risk in previously healthy children. Moreover, the majority of interactions that increased risk for PDAD were among SNPs of both hydrophilic and hydrophobic SP genes after adjusting for age, positive bacterial culture, and ventilator days. In contrast to present study of association of *SFTPA1* and *SFTPA2* with ARF, a previous study has shown that the majority of intergenic interactions associated with CF in a two-SNP model involved SNPs of *SFTPB* and *SFTPC*. Only two intergenic interactions were observed among the *SFTPA1* and *SFTPA2* SNPs [19]. Of interest, a comparison between the findings of CF and ARF shows that each hydrophobic SP gene SNPs was involved in the majority of interactions in CF, but in ARF, SNPs of the *SFTPA1* and *SFTPA2* were predominantly involved. It is not clear whether lack of a strong association of CF with innate immunity and host defense SP genes is due to the true effect of a limited role of *SFTPA1* and *SFTPA2* in CF or to limitations of the two-SNP model used in that study. Conversely, the majority of hydrophilic and hydrophobic interactions were associated with PDAD.

Based on the multiple intragenic and intergenic interactions of *SFTPA1* and *SFTPA2* observed in the present study, we speculate that innate immunity and host defense play a major role in pediatric ARF and interactions of these two genes with other SP genes play a major role in disease progression, as infection was the most common cause of ARF in our cohort (86% of ARF children had a diagnosis of RSV bronchiolitis, non-RSV bronchiolitis, and pneumonia, see Table 1). We postulate that these interactions of both hydrophilic and hydrophobic proteins together are linked to an as yet unidentified variable genetic element that has a synergistic biological role in the risk of ARF as well as its progression to PDAD.

### 4.3. Association of SNPs of SFTPA2 with ARF

The majority of intragenic and intergenic interactions (~32%) associated with increased ARF risk involve SNPs of *SFTPA2*. The two *SFTPA2* SNPs (rs1059046, rs1965708) have the highest number of interactions with other SNPs. Published studies have also shown associations of rs1059046 and rs1965708 of *SFTPA2* [42,43] with severe RSV disease. Notably, in our study 127 of the 248 children (51%) had ARF due to RSV bronchiolitis (see Table 1). Furthermore, rs1965708 is located in the carbohydrate recognition domain of SP-A2 and changes the encoded amino acid Gly/Lys at codon 223. It is plausible that this amino acid substitution decreases viral binding and consequently increases RSV load, causing ARF. However, the role (if any) of this amino acid in RSV-binding functionality has not been studied yet.

Though functions of SP-A1 and SP-A2 are not mutually exclusive, it has been shown that SP-A2 (compared to SP-A1) is better in bacterial phagocytosis and cytokine production by macrophages in response to infection and oxidative stress. [44,45,46,47]. Therefore, we speculate that ARF due to infection along with altered SP-A2 may lead to increase susceptibility of previously healthy children to ARF.

### 4.4. Association of SNPs of SFTPA1 with PDAD

In contrast to ARF, we found a significantly higher number of intragenic and intergenic interactions of *SFTPA1* (~32%) SNPs with a dominant effect to be associated with PDAD (Tables S2–S4). This indicates a role of *SFTPA1* SNPs in progression of ARF to PDAD. The *SFTPA1* SNPs (rs1136450, rs1136451) that had the highest number of interactions are located in the collagen-like domain of the SP-A1. The rs1136451 at codon 62 is silent, while rs1136450 at codon 50 changes the amino acid from Leucine to Valine. Previous animal and human studies have shown that collagen-like domain plays an important role in oligomerization and stabilization of SP-A [48]. Published studies have shown differences in oligomerization and functional capabilities of SP-A in various healthy and pulmonary disease states [49,50,51]. Furthermore, SP-A1 expressed by stably transfected CHO cells exhibits a larger size oligomers than SP-A2, and the amino acid at residue 85 within the collagen-like domain plays a key role [47]. SP-A1 has a cysteine and SP-A2 has an arginine at residue 85. Whether the amino acid 50 contributes to the oligomerization imparted by amino acid 85 remains to be determined. Moreover, SP-A1 and SP-A2 are shown to differentially affect lung function after *Klebsiella pneumoniae* infection [52], and SP-A1 is shown to exhibit higher efficiency in phospholipid adsorption and surfactant reorganization and is better (than SP-A2) in the inhibition of surfactant inactivation by serum proteins [11]. SP-A2, on the other hand, is better at enhancing bacterial phagocytosis and cytokine production by macrophages [44,45,46,47]. It is possible that the observed difference of the role of the *SFTPA1* and *SFTPA2* SNPs in PDAD and ARF, respectively, might be due to differential functions of SP-A1 and SP-A2.

### 4.5. Association of SFTPD with Decreased Risk of PDAD

We did not observe any association of *SFTPD* with ARF. Unlike in the CF study, the majority of *SFTPD* interactions were with *SFTPA1* and *SFTPA2*, and were associated with decreased risk of PDAD and all the interactions of *SFTPD* with *SFTPB* and *SFTPC* were associated with decreased risk of PDAD. Previous association studies of *SFTPD* with various pulmonary diseases have shown conflicting results, e.g., no association with ARDS [21] and Mexican COPD [53], increased risk for the positive tuberculin skin test subgroup of the Mexican tuberculosis patients [22], and decreased risk for RDS [54,55]. This apparent discrepancy is likely due to use of different patient population and study methods, i.e., the majority of the previous association studies used homogenous patient population from a single center and the single SNP method. In contrast, we have enrolled patients from 10 different centers across the United States and used two- and the three-SNP interaction model. It is known that a genetic variant in the presence of another variant can alter the susceptibility of an individual to certain diseases [56]. Moreover, human haplotype studies of SP-A and SP-D have shown a protective effect against RSV infection [23]. Therefore, it is not surprising to observe the protective effects of *SFTPD* interactions with other SP genes on PDAD. However, the biological and molecular mechanisms remain unclear and need further exploration in the future.

### 4.6. Interactions of Hydrophobic SPs (SFTPB and SFTPC) with ARF and PDAD

The hydrophobic SPs are essential for normal lung function, and SP-B polymorphisms in case-control studies have been shown to associate with neonatal RDS [17,18,57]. We observed interactions of *SFTPB* and *SFTPC* with the hydrophilic SPs (*SFTPA1*, *SFTPA2*, and *SFTPD*), but we did not identify any significant interaction among SNPs of the *SFTPB* and *SFTPC* alone in ARF or PDAD. A previous study of rs1130866 of *SFTPB* interaction with *SFTPA1* and *SFTPA2* showed association with neonatal RDS in Finnish children [57]. This is in line with our results, where SNP–SNP interactions among *SFTPB, SFTPA1,* and *SFTPA2* associate with ARF and PDAD. Interestingly, we observed that interactions of *SFTPB* and *SFTPC* SNPs are associated with decreased ARF risk, but increased risk of PDAD. The differential impact of these interactions on the disease and its progression further highlights the role of complexity of SP genetics in acute and chronic pulmonary diseases and importance of studying the SNP–SNP interaction instead of a single-SNP association study.

### 4.7. Haplotype Association with ARF Risk

Haplotypes consist of SNPs that are inherited together and shown to be a powerful tool to study associations of polymorphisms in complex diseases such as ARF [58]. We found a haplotype “GG” Val50(G)-Pro62(G) within *SFTPA1* to have a dominant effect on ARF risk (OR = 9.3). Previously, the *SFPTA1* and *SFTPA2* haplotype 6A^3^/1A^1^ has been shown to associate with severe RSV in Finnish children [42]. Multiple studies have shown an association of SP-A1 and SP-A2 gene haplotypes with adult ARDS [21], IPF [33], neonatal RDS [16], and COPD [53]. Of relevance, a previous study from our group has shown a protective effect of haplotype “DA160_A/SP-A2 1A^5^”of *SFTPD* and *SFTPA2,* respectively, on RSV disease [23]. However, we did not find haplotypes of *SFTPD* and *SFTPA2* to associate with ARF risk. Instead, our study showed a haplotype “AA” 138Asn-186Asn, consisting of rs4715 Asn(A) at codon 138 and rs1124 Asn(A) at codon 186 within the *SFTPC* gene to associate with lower risk for ARF (OR = 0.4). In contrast to our study, the 138Asn-186Asn haplotype was associated with increased RDS risk in Finnish neonates [40]. Moreover, Puthothu et al. has shown that the 138Thr-186Asn, and not the 138Asn-186Asn haplotype was overrepresented in German children with severe RSV bronchiolitis [59] but protective for bronchial asthma [59]. Although 51% of our study cohort had RSV bronchiolitis, other infectious and non-infectious etiologies responsible for ARF may play a role. The observed difference between the study findings may be explained by differences in study design, sample population, and statistical methods used for analysis.

The strengths of the study include (a) its multicenter prospective trial design, enrolling previously healthy children, (b) use of a robust and novel statistical approach of a single-, two-, and three-SNP model and multiple comparisons adjusting for variables, and (c) study of SNPs of physiologically relevant SP genes to ARF and PDAD. Our study has few limitations: (a) use of newborn controls instead of age-matched controls. For our study, we enrolled young (average age of the study cohort ~ 3 months) previously healthy children from 10 different sites representing the United States population. Therefore, we believe that the findings of the current study will have a high clinical impact if the results are replicated in other sample sets; (b) lack of biological data such as surfactant protein concentration in BAL of study cohort and its impact on SNP–SNP interactions. It has been shown that ARF is associated with surfactant dysfunction along with dysregulated inflammation [2]. Moreover, previous studies of exogenous surfactant for ARF treatment yielded conflicting results [60,61], perhaps due to absence of SP-A in exogenous surfactant. Our findings indicate that SP-A may play a major role in ARF pathogenesis secondary to infection mediated direct lung injury and should be considered for future therapies for ARF. In the future, clinicians may use these interactions as a marker for early identification of children at risk for ARF and its morbidity, thereby allowing for an earlier initiation of specific treatment such as exogenous surfactant to reduce either the acute symptoms or long-term pulmonary sequelae.

In conclusion, we demonstrated for the first time that associations of SP gene polymorphisms with ARF and its progression to PDAD in children. Based on the collective information and biological plausibility, SP genes, particularly the innate immunity and host defense genes, are likely to be gene modifiers in respiratory infection that induces ARF in healthy children and they must be studied further.

## Figures and Tables

**Table 1:** Demographics of cohort stratified by pulmonary dysfunction at discharge (PDAD).

Variable	No PDAD (*n* = 162)	PDAD (*n* = 86)	*p* value
Demographics Age (months) Female/male (%/%)	2.8 ± 3.5 63/99 (39/61)	5.4 ± 5.6 36/50 (42/58)	<0.001 0.89
Race White Black Mixed/Other	129 (80) 23 (14) 10 (6)	66 (77) 15 (17) 5 (6)	0.74
Ethnicity Hispanic Non-Hispanic	31 (19) 131 (81)	21 (24) 65 (76)	0.61
Admission diagnosis (%) RSV bronchiolitis Other bronchiolitis Other pneumonia Other respiratory failure Non-pulmonary	89 (55) 39 (24) 13 (8) 17 (10) 4 (2)	38 (44) 17 (20) 17 (20) 12 (14) 2 (2)	0.08
PRISM III score	4.6 ± 3.6	4.8 ± 4.0	0.69
Specific virus (%) (*n* = 187) RSV Influenza Parainfluenza Adenovirus	102 (79) 3 (2) 1 (1) 1 (1)	38 (64) 1 (1) 2 (2) 2 (3)	0.03 1 0.33 0.24
Positive bacterial culture (%)(*n* = 195)	81 (63)	54 (82)	0.009
Duration of support Ventilator days Oxygen days PICU days	6.5 ± 4.0 10.0 ± 5.1 8.3 ± 4.6	9.5 ± 12.1 12.7 ± 14.5 12.1 ± 13.5	0.004 0.03 0.001

PRISM III score-Pediatric Risk of Mortality III score, RSV-respiratory syncytial virus.

**Table 2:** Association of SNPs of SP genes with ARF compared to newborn controls after adjusting for sex and ethnicity.

	Gene	SNP #1 ID	Gene	SNP #2 ID	Gene	SNP #3 ID	Interaction	*p* value	FDR	OR (95% CI)
**One dominant effect**										
1 *	*SFTPA2*	rs1059046	*SFTPA2*	rs1965707	*SFTPA1*	rs1059047	a1 × d2 × a3	0.001285994	0.001	2.2 (1.3–3.6)
2 *	*SFTPA2*	rs1059046	*SFTPA2*	rs1965707	*SFTPA1*	rs1136451		0.001052925	0.00105	2.2 (1.3–3.7)
3 *	*SFTPA2*	rs1059046	*SFTPA2*	rs1965707	*SFTPA1*	rs4253527		0.000833915	0.00083	2.3 (1.4–4.0)
4 *	*SFTPA2*	rs1059046	*SFTPA2*	rs1965708	*SFTPA1*	rs1136451		0.000662744	0.00066	2.6 (1.4–4.7)
5 #^a^	*SFTPA2*	rs1059046	*SFTPA2*	rs17886395	*SFTPA2*	rs1965708	a1 × a2 × d3	0.000229167	0.00020	0.4 (0.2–0.7)
**Two dominant effect**										
1 *^a^	*SFTPA2*	rs1059046	*SFTPA2*	rs17886395	*SFTPA2*	rs1965707	a1 × d2 × d3	0.00008108	0.00008	2.1 (1.4–3.2)
2 *^a^	*SFTPA2*	rs1059046	*SFTPA2*	rs17886395	*SFTPA2*	rs1965708		0.001549286	0.00155	1.8 (1.2–2.7)
3 *	*SFTPA2*	rs1059046	*SFTPA2*	rs1965707	*SFTPA1*	rs1059047		0.00341059	0.00341	1.8 (1.2–2.7)
4 *	*SFTPA2*	rs1059046	*SFTPA2*	rs1965707	*SFTPA1*	rs1136451		0.002675982	0.00268	1.8 (1.2–2.7)
5 *	*SFTPA2*	rs1059046	*SFTPA2*	rs1965708	*SFTPA1*	rs1059047		0.000228776	0.00023	2.2 (1.4–3.5)
6 *	*SFTPA2*	rs1059046	*SFTPA1*	rs1059047	*SFTPA1*	rs1136450		0.001111869	0.00111	2.1 (1.3–3.3)
7 #	*SFTPA2*	rs1965707	*SFTPA2*	rs1965708	*SFTPC*	rs4715		0.00053651	0.00050	0.3 (0.1–0.6)
8 #^a^	*SFTPA1*	rs1059047	*SFTPA1*	rs1136451	*SFTPA1*	rs4253527		0.004439716	0.00400	0.3 (0.1–0.8)
9 *^a^	*SFTPA2*	rs1059046	*SFTPA2*	rs17886395	*SFTPA2*	rs1965707	d1 × a2 × d3	0.000424175	0.00042	1.8 (1.3–2.5)
10 *^a^	*SFTPA2*	rs1059046	*SFTPA2*	rs17886395	*SFTPA2*	rs1965708		0.001836416	0.00184	1.7 (1.2–2.3)
11 *	*SFTPA2*	rs17886395	*SFTPA1*	rs1136450	*SFTPC*	rs4715		0.000648071	0.00049	4.0 (1.7–10.1)
12 #	*SFTPA2*	rs17886395	*SFTPA1*	rs1059047	*SFTPA1*	rs1136450		0.000490381	0.00060	0.6 (0.5–0.8)
13 *	*SFTPA2*	rs17886395	*SFTPA2*	rs1965707	*SFTPA1*	rs1136450	d1 × d2 × a3	0.000191969	0.00019	2.2 (1.4–3.4)
14 #	*SFTPA2*	rs17886395	*SFTPA1*	rs1136450	*SFTPC*	rs4715		0.000158449	0.00010	0.2 (0.1–0.5)
**Three dominant effect**										
1 *	*SFTPA2*	rs17886395	*SFTPA1*	rs1136451	*SFTPA1*	rs4253527	d1 × d2 × d3	0.000333494	0.00005	1.6 (1.3–2.1)
2 #^a^	*SFTPA2*	rs1059046	*SFTPA2*	rs17886395	*SFTPA2*	rs1965708		0.005639344	0.00030	0.6 (0.5–0.8)
3 #	*SFTPA2*	rs1059046	*SFTPA2*	rs1965707	*SFTPC*	rs4715		0.004814545	0.00600	0.6 (0.4–0.9)
4 #	*SFTPA2*	rs1059046	*SFTPA1*	rs1059047	*SFTPA1*	rs1136450		0.00000000002	0.00500	0.7 (0.6–0.9)
5 #	*SFTPA2*	rs1059046	*SFTPA1*	rs1136450	*SFTPC*	rs4715		0.00336572	0.00000	0.3 (0.2–0.4)
6 #	*SFTPA2*	rs1059046	*SFTPA1*	rs1136451	*SFTPC*	rs4715		0.000000227	0.00300	0.5 (0.4–0.8)
7 #^a^	*SFTPA2*	rs17886395	*SFTPA2*	rs1965707	*SFTPA2*	rs1965708		0.000004297	0.00000	0.5 (0.4–0.7)
8 #	*SFTPA2*	rs17886395	*SFTPA1*	rs1059047	*SFTPA1*	rs1136450		0.000809901	0.00000	0.6 (0.5–0.7)
9 #	*SFTPA2*	rs17886395	*SFTPA1*	rs1059047	*SFTPA1*	rs1136451		0.000054837	0.00080	0.7 (0.5–0.9)
10 #	*SFTPA2*	rs17886395	*SFTPA1*	rs1136451	*SFTPC*	rs4715		0.000094992	0.00009	0.4 (0.3–0.7)
11 #	*SFTPA2*	rs17886395	*SFTPA1*	rs4253527	*SFTPC*	rs4715		0.00329539	0.00300	0.5 (0.3–0.8)
12 #	*SFTPA2*	rs1965707	*SFTPA2*	rs1965708	*SFTPA1*	rs1136450		0.000141004	0.00010	0.7 (0.5–0.8)
13 #	*SFTPA2*	rs1965707	*SFTPA1*	rs1136450	*SFTPC*	rs4715		0.003330483	0.00300	0.6 (0.4–0.8)
14 #	*SFTPA1*	rs1059047	*SFTPA1*	rs1136450	*SFTPA1*	rs1136451		0.005263705	0.00500	0.7 (0.6–0.9)
15 #	*SFTPA1*	rs1136451	*SFTPA1*	rs4253527	*SFTPC*	rs4715		0.00126707	0.00100	0.5 (0.3–0.8)

^a^ intragenic interactions. Sign “*” and “#” show interactions that increased and decreased ARF risk, respectively. FDR – False discovery rate.

## Supplementary Materials

The following are available online at https://www.mdpi.com/2077-0383/9/4/1183/s1, Table S1: Demographics of the cohort of ARF and newborn control, Table S2: Association of SP genes with PDAD subgroup after adjusting for age, Table S3: Association of SP genes with PDAD subgroup after adjusting for positive bacterial culture, Table S4: Association of SP genes with PDAD subgroup after adjusting for ventilator days.
