# Supplementary material for: Association of SNP–SNP Interactions of Surfactant Protein Genes with Pediatric Acute Respiratory Failure
Source: J Clin Med. 2020 Apr 20;9(4):1183. doi: 10.3390/jcm9041183 (PMC7231046; doi:10.3390/jcm9041183)
Supplement: Supplementary file 1 [file jcm-09-01183-s001.pdf]

Supplementary Table 1: Demographics of the cohort of ARF and newborn control

**Demographics**	**ARF (n = 248)**	**Newborn control (n = 468)**	**p value**
**Sex:**			0.000
Female/male (%/%)	99/149 (39/61)	240/228 (51/49)	
**Race:**			0.6
White	197 (79)	375 (80)	
Black	42 (17)	86 (18)	
Asian	5 (2)	-	
Native Hawaiian or Pacific Islanders	1 (1)	1 (1)	
American Indian/Alaska Native	3 (1)	-	
Mixed/Other	-	6 (1)	
**Ethnicity:**			0.000
Hispanic	52 (21)	3 (1)	
Non-Hispanic	196 (79)	459 (98)	
Mixed	-	6 (1)	
**Admission diagnosis (%):**		-	
RSV bronchiolitis	127 (51)		
Other bronchiolitis	56 (23)		
Other pneumonia	30 (12)		
Other respiratory failure	29 (12)		
Non-pulmonary	6 (2)		
PRISM III score	4.6 ±3.7	-	
Specific virus (%)(n=187)		-	
RSV	140 (75)		
Influenza	4 (2)		
Parainfluenza	3 (1)		
Adenovirus	2 (1)		
Positive bacterial culture (%)(n=195)	135 (70)	-	
Duration of support		-	
Ventilator days	7.5 ±7.9		
Oxygen days	10.9 ±9.3		
PICU days	9.6 ±9.0		

ARF - acute respiratory failure, RSV – respiratory syncytial virus, PICU – pediatric intensive care unit

Supplementary Table 2 Association of SP genes with PDAD subgroup after adjusting for age

	Gene	SNP #1 ID	Gene	SNP #2 ID	Gene	SNP #3 ID	Interaction	Adjusted p value (FDR)	OR (95% CI)
**Two dominant effect**									
1*	*SFTPA1*	rs1136451	*SFTPB*	rs1130866	*SFTPD*	rs721917	d1 X d2 X a3	0.04557	3.1 (1.7-5.9)
**Three dominant effect**									
1*[a]	*SFTPA2*	rs1059046	*SFTPA1*	rs1136450	*SFTPA2*	rs1965707	d1 X d2 X d3	0.02951	1.7 (1.2-2.3)
2#[a]	*SFTPA2*	rs1059046	*SFTPA1*	rs1136450	*SFTPD*	rs2243639		0.01682	0.6 (0.4-0.8)
3*	*SFTPA2*	rs1059046	*SFTPA1*	rs1136451	*SFTPC*	rs1124		0.02797	1.7 (1.2-2.4)
4*[a]	*SFTPA2*	rs1059046	*SFTPA2*	rs17886395	*SFTPD*	rs721917		0.02797	1.7 (1.2-2.3)
5#[a]	*SFTPA2*	rs1059046	*SFTPA2*	rs1965707	*SFTPD*	rs721917		0.02797	0.6 (0.4-0.8)
6#[a]	*SFTPA2*	rs1059046	*SFTPA2*	rs1965708	*SFTPD*	rs721917		0.00018	0.5 (0.3-0.6)
7*[a]	*SFTPA2*	rs1059046	*SFTPD*	rs721917	*SFTPD*	rs2243639		0.00005	2.3 (1.7-3.3)
8#	*SFTPA2*	rs1059046	*SFTPD*	rs2243639	*SFTPC*	rs1124		0.00195	0.5 (0.4-0.7)
9#	*SFTPA1*	rs1059047	*SFTPA1*	rs1136450	*SFTPB*	rs1130866		0.03483	0.6 (0.4-0.8)
10#	*SFTPA1*	rs1059047	*SFTPA1*	rs1136451	*SFTPC*	rs4715		0.03086	0.6 (0.4-0.8)
11#[a]	*SFTPA1*	rs1059047	*SFTPA1*	rs1059057	*SFTPD*	rs2243639		0.03757	0.6 (0.4-0.8)
12#	*SFTPA1*	rs1059047	*SFTPD*	rs721917	*SFTPB*	rs1130866		0.01682	0.6 (0.4-0.8)
13#	*SFTPA1*	rs1059047	*SFTPD*	rs721917	*SFTPC*	rs4715		0.01116	0.5 (0.4-0.8)
14#	*SFTPA1*	rs1059047	*SFTPD*	rs2243639	*SFTPC*	rs4715		0.00586	0.5 (0.3-0.7)
15#	*SFTPA1*	rs1059057	*SFTPD*	rs721917	*SFTPB*	rs1130866		0.03086	0.6 (0.4-0.8)
16#	*SFTPA1*	rs1059057	*SFTPD*	rs721917	*SFTPC*	rs4715		0.01479	0.5 (0.4-0.8)
17#	*SFTPA1*	rs1059057	*SFTPD*	rs2243639	*SFTPC*	rs4715		0.02797	0.5 (0.4-0.8)
18#	*SFTPA1*	rs1136450	*SFTPA1*	rs1059057	*SFTPB*	rs1130866		0.0301	0.6 (0.4-0.8)
19#	*SFTPA1*	rs1136450	*SFTPA2*	rs1965708	*SFTPC*	rs1124		0.03483	0.6 (0.4-0.9)
20#	*SFTPA1*	rs1136450	*SFTPD*	rs721917	*SFTPC*	rs4715		0.00267	0.5 (0.4-0.7)
21#	*SFTPA1*	rs1136450	*SFTPD*	rs2243639	*SFTPC*	rs1124		0.01207	0.6 (0.4-0.8)
22*	*SFTPA1*	rs1136450	*SFTPB*	rs1130866	*SFTPC*	rs4715		0.00802	1.8 (1.3-2.6)

23#	*SFTPA1*	rs1136451	*SFTPA2*	rs17886395	*SFTPB*	rs1130866		0.01682	0.5 (0.4-0.8)
24#	*SFTPA1*	rs1136451	*SFTPA1*	rs1059057	*SFTPC*	rs4715		0.03792	0.6 (0.4-0.8)
25*	*SFTPA1*	rs1136451	*SFTPA2*	rs1965707	*SFTPC*	rs1124		0.00771	1.9 (1.3-2.8)
26*	*SFTPA1*	rs1136451	*SFTPA1*	rs4253527	*SFTPC*	rs4715		0.00063	2.3 (1.5-3.5)
27*	*SFTPA2*	rs17886395	*SFTPA1*	rs4253527	*SFTPC*	rs4715		0.00028	2.4 (1.6-3.6)
28#[a]	*SFTPA2*	rs17886395	*SFTPA2*	rs1965708	*SFTPD*	rs721917		0.00586	0.5 (0.3-0.7)
29#	*SFTPA2*	rs1965707	*SFTPD*	rs2243639	*SFTPC*	rs4715		0.00018	0.4 (0.3-0.6)
30#	*SFTPA2*	rs1965707	*SFTPD*	rs2243639	*SFTPC*	rs1124		0	0.4 (0.3-0.5)
31#	*SFTPA2*	rs1965707	*SFTPB*	rs1130866	*SFTPC*	rs1124		0	0.4 (0.3-0.5)
32#	*SFTPA2*	rs1965708	*SFTPD*	rs2243639	*SFTPC*	rs4715		0.00007	0.4 (0.3-0.6)
33#	*SFTPA2*	rs1965708	*SFTPD*	rs2243639	*SFTPC*	rs1124		0.00018	0.4 (0.3-0.6)
34#	*SFTPA2*	rs1965708	*SFTPB*	rs1130866	*SFTPC*	rs1124		0	0.4 (0.3-0.5)
35#	*SFTPA1*	rs4253527	*SFTPA2*	rs1965708	*SFTPB*	rs1130866		0.02797	0.5 (0.4-0.8)
36*	*SFTPA1*	rs4253527	*SFTPD*	rs721917	*SFTPB*	rs1130866		0.01543	1.8 (1.3-2.6)
37#	*SFTPD*	rs721917	*SFTPC*	rs4715	*SFTPC*	rs1124		0.03483	0.6 (0.5-0.9)

[a] Interactions among the SNPs of hydrophilic SP SNPs Sign “*” and “#” show interactions that increased and decreased PDAD risk, respectively.
FDR – False discovery rate

Supplementary Table 3 Association of SP genes with PDAD subgroup after adjusting for positive bacterial culture

	Gene	SNP #1 ID	Gene	SNP #2 ID	Gene	SNP #3 ID	Interaction	Adjusted p value (FDR)	OR (95% CI)
**Two dominant effect**									
1*	*SFTPA1*	rs1059047	*SFTPD*	rs721917	*SFTPB*	rs1130866	d1 x d2 x a3	0.036495384	3.7 (1.7-8.2)
2*	*SFTPA1*	rs1136450	*SFTPA1*	rs1059057	*SFTPC*	rs4715		0.036495384	3.2 (1.6-6.4)
3#	*SFTPA1*	rs1136450	*SFTPA1*	rs1965707	*SFTPC*	rs4715		0.000446136	0.2 (0.1-0.4)
4*	*SFTPA1*	rs1136451	*SFTPD*	rs721917	*SFTPB*	rs1130866		0.007650966	4.1 (2.0-8.7)
5*	*SFTPA1*	rs1059047	*SFTPB*	rs1130866	*SFTPC*	rs1124	a1 x d2 x d3	0.009142874	2.7 (1.6-4.5)
6*	*SFTPA1*	rs1136450	*SFTPA1*	rs1136451	*SFTPB*	rs1130866		0.021999602	3.4 (1.6-7.3)
7*	*SFTPA1*	rs1136450	*SFTPD*	rs2243639	*SFTPC*	rs4715		0.039800503	3.1 (1.5-6.4)
8*	*SFTPA1*	rs1136451	*SFTPB*	rs1130866	*SFTPC*	rs1124		0.0013801	3.3 (1.9-5.7)
9*	*SFTPA2*	rs17886395	*SFTPB*	rs1130866	*SFTPC*	rs1124		0.009525117	2.8 (1.6-5.0)
10*	*SFTPA2*	rs1059057	*SFTPB*	rs1130866	*SFTPC*	rs1124		0.015321414	2.5 (1.5-4.3)
11*	*SFTPA1*	rs4253527	*SFTPB*	rs1130866	*SFTPC*	rs1124		0.000202956	3.6 (2.1-6.2)
12#	*SFTPA1*	rs1136450	*SFTPD*	rs721917	*SFTPB*	rs1130866	d1 x a2 x d3	0.002561308	0.2 (0.1-0.5)
13*	*SFTPA1*	rs4253527	*SFTPD*	rs721917	*SFTPC*	rs4715		0.039821764	4.0 (1.8-9.5)
**Three dominant effect**									
1#[b]	*SFTPA2*	rs1059046	*SFTPA1*	rs1136450	*SFTPA1*	rs1059057	d1 x d2 x d3	0.0348	0.6 (0.4-0.9)
2*[b]	*SFTPA2*	rs1059046	*SFTPA1*	rs1136450	*SFTPA2*	rs1965707		0.0084	1.9 (1.3-2.8)
3#[b]	*SFTPA2*	rs1059046	*SFTPA1*	rs1136450	*SFTPD*	rs2243639		0.0179	0.6 (0.4-0.8)
4#	*SFTPA2*	rs1059046	*SFTPA1*	rs1136450	*SFTPC*	rs1124		0.0079	0.5 (0.4-0.8)
5*[b]	*SFTPA2*	rs1059046	*SFTPD*	rs721917	*SFTPD*	rs2243639		0.0138	1.9 (1.3-2.8)
6#	*SFTPA2*	rs1059046	*SFTPD*	rs2243639	*SFTPC*	rs1124		0.0453	0.6 (0.4-0.9)
7#	*SFTPA1*	rs1059047	*SFTPA1*	rs1136450	*SFTPB*	rs1130866		0.0453	0.6 (0.4-0.9)
8#[a]	*SFTPA1*	rs1059047	*SFTPA1*	rs1136451	*SFTPA1*	rs4253527		0.0138	0.0 (0.0-0.5)
9#	*SFTPA1*	rs1059047	*SFTPA1*	rs1136451	*SFTPC*	rs4715		0.0028	0.4 (0.3-0.7)
10#	*SFTPA1*	rs1059047	*SFTPA1*	rs1136451	*SFTPC*	rs1124		0.0090	0.5 (0.3-0.7)

11#[b]	*SFTPA1*	rs1059047	*SFTPA1*	rs1059057	*SFTPD*	rs721917	0.0138	0.5 (0.3-0.8)
12#[b]	*SFTPA1*	rs1059047	*SFTPA1*	rs1059057	*SFTPD*	rs2243639	0.0157	0.5 (0.3-0.8)
13#	*SFTPA1*	rs1059047	*SFTPA2*	rs1965707	*SFTPB*	rs1130866	0.0157	0.5 (0.3-0.8)
14#	*SFTPA1*	rs1059047	*SFTPD*	rs721917	*SFTPC*	rs4715	0.0018	0.4 (0.3-0.6)
15#	*SFTPA1*	rs1059047	*SFTPD*	rs2243639	*SFTPC*	rs4715	0.0032	0.4 (0.3-0.7)
16*	*SFTPA1*	rs1059047	*SFTPB*	rs1130866	*SFTPC*	rs1124	0.0042	2.2 (1.4-3.4)
17*[a]	*SFTPA1*	rs1136450	*SFTPA1*	rs1136451	*SFTPA1*	rs4253527	0.0017	2.5 (1.6-3.9)
18#[b]	*SFTPA1*	rs1136450	*SFTPA1*	rs1136451	*SFTPD*	rs2243639	0.0138	0.5 (0.3-0.8)
19#	*SFTPA1*	rs1136450	*SFTPA1*	rs1059057	*SFTPB*	rs1130866	0.0360	0.6 (0.4-0.9)
20*[b]	*SFTPA1*	rs1136450	*SFTPA2*	rs1965707	*SFTPA2*	rs1965708	0.0084	2.0 (1.3-3.0)
21*[b]	*SFTPA1*	rs1136450	*SFTPA2*	rs1965708	*SFTPD*	rs2243639	0.0226	1.8 (1.2-2.8)
22#	*SFTPA1*	rs1136450	*SFTPA2*	rs1965708	*SFTPC*	rs1124	0.0073	0.5 (0.3-0.7)
23#[b]	*SFTPA1*	rs1136451	*SFTPA2*	rs17886395	*SFTPD*	rs2243639	0.0338	0.6 (0.4-0.8)
24#	*SFTPA1*	rs1136451	*SFTPA2*	rs17886395	*SFTPB*	rs1130866	0.0134	0.5 (0.3-0.8)
25*[b]	*SFTPA1*	rs1136451	*SFTPA1*	rs1059057	*SFTPA2*	rs1965707	0.0214	2.0 (1.3-3.2)
26#[b]	*SFTPA1*	rs1136451	*SFTPA1*	rs1059057	*SFTPD*	rs721917	0.0275	0.5 (0.3-0.8)
27#	*SFTPA1*	rs1136451	*SFTPA1*	rs1059057	*SFTPC*	rs4715	0.0046	0.4 (0.3-0.7)
28#	*SFTPA1*	rs1136451	*SFTPA1*	rs1059057	*SFTPC*	rs1124	0.0145	0.5 (0.3-0.8)
29#	*SFTPA1*	rs1136451	*SFTPA2*	rs1965707	*SFTPB*	rs1130866	0.0490	0.6 (0.4-0.9)
30*	*SFTPA1*	rs1136451	*SFTPA1*	rs4253527	*SFTPC*	rs4715	0.0145	2.0 (1.3-3.2)
31#	*SFTPA1*	rs1136451	*SFTPA2*	rs1965708	*SFTPB*	rs1130866	0.0364	0.5 (0.4-0.8)
32#	*SFTPA1*	rs1136451	*SFTPD*	rs2243639	*SFTPC*	rs4715	0.0063	0.5 (0.3-0.7)
33#[b]	*SFTPA2*	rs17886395	*SFTPA1*	rs1059057	*SFTPD*	rs2243639	0.0309	0.5 (0.3-0.8)
34#	*SFTPA2*	rs17886395	*SFTPA1*	rs1059057	*SFTPC*	rs4715	0.0145	0.5 (0.3-0.8)
35*	*SFTPA2*	rs17886395	*SFTPA1*	rs4253527	*SFTPC*	rs4715	0.0050	2.2 (1.4-3.6)
36#[b]	*SFTPA2*	rs17886395	*SFTPA2*	rs1965708	*SFTPD*	rs721917	0.0013	0.4 (0.3-0.6)
37#	*SFTPA2*	rs17886395	*SFTPD*	rs2243639	*SFTPC*	rs4715	0.0011	0.4 (0.3-0.6)
38#	*SFTPA2*	rs17886395	*SFTPD*	rs2243639	*SFTPC*	rs1124	0.0170	0.5 (0.4-0.8)

39#	*SFTPA1*	rs1059057	*SFTPA2*	rs1965707	*SFTPB*	rs1130866		0.0138	0.5 (0.3-0.8)
40*	*SFTPA1*	rs1059057	*SFTPA2*	rs1965707	*SFTPC*	rs1124		0.0214	1.9 (1.2-3.1)
41#	*SFTPA1*	rs1059057	*SFTPD*	rs721917	*SFTPC*	rs4715		0.0032	0.4 (0.3-0.7)
42#	*SFTPA1*	rs1059057	*SFTPD*	rs2243639	*SFTPC*	rs4715		0.0275	0.5 (0.3-0.8)
43*	*SFTPA1*	rs1059057	*SFTPB*	rs1130866	*SFTPC*	rs1124		0.0226	1.8 (1.2-2.8)
44#	*SFTPA2*	rs1965707	*SFTPA2*	rs1965708	*SFTPC*	rs1124		0.0191	0.6 (0.4-0.8)
45#	*SFTPA2*	rs1965707	*SFTPD*	rs2243639	*SFTPC*	rs4715		0.0013	0.4 (0.3-0.6)
46#	*SFTPA2*	rs1965707	*SFTPD*	rs2243639	*SFTPC*	rs1124		0.0001	0.4 (0.2-0.6)
47#	*SFTPA2*	rs1965707	*SFTPB*	rs1130866	*SFTPC*	rs1124		0.0001	0.4 (0.3-0.6)
48#	*SFTPA1*	rs4253527	*SFTPA2*	rs1965708	*SFTPB*	rs1130866		0.0138	0.5 (0.3-0.8)
49*	*SFTPA1*	rs4253527	*SFTPD*	rs721917	*SFTPB*	rs1130866		0.0171	1.9 (1.3-2.9)
50#	*SFTPA1*	rs4253527	*SFTPD*	rs2243639	*SFTPC*	rs4715		0.0011	0.4 (0.2-0.6)
51#	*SFTPA1*	rs4253527	*SFTPD*	rs2243639	*SFTPC*	rs1124		0.0032	0.4 (0.3-0.7)
52*	*SFTPA1*	rs4253527	*SFTPB*	rs1130866	*SFTPC*	rs1124		0.0001	2.7 (1.8-4.1)
53#	*SFTPA2*	rs1965708	*SFTPD*	rs2243639	*SFTPC*	rs4715		0.0090	0.5 (0.3-0.8)
54#	*SFTPA2*	rs1965708	*SFTPD*	rs2243639	*SFTPC*	rs1124		0.0176	0.5 (0.3-0.8)
55#	*SFTPA2*	rs1965708	*SFTPB*	rs1130866	*SFTPC*	rs1124		0.0001	0.4 (0.2-0.6)
56#	*SFTPD*	rs721917	*SFTPC*	rs4715	*SFTPC*	rs1124		0.0138	0.6 (0.4-0.8)
57#	*SFTPD*	rs2243639	*SFTPC*	rs4715	*SFTPC*	rs1124		0.0035	0.5 (0.3-0.7)

[a] shows intragenic interactions

[b] Interactions among the SNPs of hydrophilic SP SNPs

Sign “*” and “#” show interactions that increased and decreased PDAD risk, respectively.

FDR – False discovery rate

Supplementary Table 4 Association of SP genes with PDAD subgroup after adjusting for ventilator days

	Gene	SNP #1 ID	Gene	SNP #2 ID	Gene	SNP #3 ID	Interaction	Adjusted p value (FDR)	OR (95% CI)
**Three dominant effect**									
1*[b]	*SFTPA2*	rs1059046	*SFTPA1*	rs1136450	*SFTPA2*	rs1965707	d1 x d2 x d3	0.01279	1.7 (1.2-2.4)
2#[b]	*SFTPA2*	rs1059046	*SFTPA1*	rs1136450	*SFTPD*	rs2243639		0.00487	0.5 (0.4-0.8)
3*	*SFTPA2*	rs1059046	*SFTPA1*	rs1136451	*SFTPC*	rs1124		0.03323	1.7 (1.2-2.4)
4*[b]	*SFTPA2*	rs1059046	*SFTPA2*	rs17886395	*SFTPD*	rs721917		0.03506	1.6 (1.2-2.2)
5#[b]	*SFTPA2*	rs1059046	*SFTPA2*	rs1965707	*SFTPD*	rs721917		0.01405	0.6 (0.4-0.8)
6*	*SFTPA2*	rs1059046	*SFTPA1*	rs4253527	*SFTPC*	rs1124		0.03207	1.7 (1.2-2.5)
7#[b]	*SFTPA2*	rs1059046	*SFTPA2*	rs1965708	*SFTPD*	rs721917		0.00020	0.5 (0.3-0.7)
8*[b]	*SFTPA2*	rs1059046	*SFTPD*	rs721917	*SFTPD*	rs2243639		0.00105	2.0 (1.4-2.8)
9#	*SFTPA2*	rs1059046	*SFTPD*	rs2243639	*SFTPC*	rs1124		0.00180	0.5 (0.4-0.7)
10#	*SFTPA1*	rs1059047	*SFTPA1*	rs1136450	*SFTPB*	rs1130866		0.01638	0.6 (0.4-0.8)
11#[b]	*SFTPA1*	rs1059047	*SFTPA1*	rs1136451	*SFTPD*	rs4253527		0.03352	0.0 (0.0-0.6)
12#	*SFTPA1*	rs1059047	*SFTPA1*	rs1136451	*SFTPC*	rs4715		0.03323	0.6 (0.4-0.8)
13#	*SFTPA1*	rs1059047	*SFTPA1*	rs1136451	*SFTPC*	rs1124		0.03707	0.6 (0.4-0.9)
14#[b]	*SFTPA1*	rs1059047	*SFTPA1*	rs1059057	*SFTPD*	rs2243639		0.03323	0.6 (0.4-0.8)
15#	*SFTPA1*	rs1059047	*SFTPD*	rs721917	*SFTPB*	rs1130866		0.03626	0.6 (0.4-0.9)
16#	*SFTPA1*	rs1059047	*SFTPD*	rs721917	*SFTPC*	rs4715		0.01279	0.5 (0.4-0.8)
17#	*SFTPA1*	rs1059047	*SFTPD*	rs2243639	*SFTPC*	rs4715		0.03352	0.6 (0.4-0.8)
18*[a]	*SFTPA1*	rs1136450	*SFTPA1*	rs1136451	*SFTPA1*	rs4253527		0.04196	1.7 (1.2-2.5)
19#	*SFTPA1*	rs1136450	*SFTPA2*	rs17886395	*SFTPB*	rs1130866		0.03626	0.6 (0.4-0.9)
20#	*SFTPA1*	rs1136450	*SFTPA1*	rs1059057	*SFTPB*	rs1130866		0.01638	0.6 (0.4-0.8)
21#	*SFTPA1*	rs1136450	*SFTPA1*	rs1059057	*SFTPC*	rs4715		0.03707	0.6 (0.4-0.9)
22#	*SFTPA1*	rs1136450	*SFTPA2*	rs1965708	*SFTPC*	rs1124		0.03390	0.6 (0.4-0.9)
23#	*SFTPA1*	rs1136450	*SFTPD*	rs721917	*SFTPC*	rs4715		0.01405	0.6 (0.4-0.8)

24#	*SFTPA1*	rs1136450	*SFTPD*	rs721917	*SFTPC*	rs1124		0.03626	0.6 (0.5-0.9)
25#	*SFTPA1*	rs1136450	*SFTPD*	rs2243639	*SFTPC*	rs1124		0.00475	0.5 (0.4-0.7)
26*	*SFTPA1*	rs1136450	*SFTPB*	rs1130866	*SFTPC*	rs4715		0.02838	1.7 (1.2-2.3)
27#[b]	*SFTPA1*	rs1136451	*SFTPA2*	rs17886395	*SFTPD*	rs2243639		0.03323	0.6 (0.4-0.8)
28#	*SFTPA1*	rs1136451	*SFTPA2*	rs17886395	*SFTPB*	rs1130866		0.02972	0.6 (0.4-0.8)
29*	*SFTPA1*	rs1136451	*SFTPA2*	rs1965707	*SFTPC*	rs1124		0.00305	2.0 (1.4-2.9)
30*	*SFTPA1*	rs1136451	*SFTPA1*	rs4253527	*SFTPC*	rs4715		0.00178	2.2 (1.5-3.3)
31*	*SFTPA2*	rs17886395	*SFTPA1*	rs4253527	*SFTPC*	rs4715		0.00015	2.5 (1.7-3.8)
32#[b]	*SFTPA2*	rs17886395	*SFTPA2*	rs1965708	*SFTPD*	rs721917		0.02185	0.6 (0.4-0.8)
33#	*SFTPA1*	rs1059057	*SFTPD*	rs721917	*SFTPC*	rs4715		0.01405	0.5 (0.4-0.8)
34#	*SFTPA2*	rs1965707	*SFTPA2*	rs1965708	*SFTPC*	rs1124		0.03323	0.6 (0.4-0.8)
35#	*SFTPA2*	rs1965707	*SFTPD*	rs2243639	*SFTPC*	rs4715		0.00105	0.5 (0.3-0.7)
36#	*SFTPA2*	rs1965707	*SFTPD*	rs2243639	*SFTPC*	rs1124		0.00000	0.4 (0.3-0.6)
37#	*SFTPA2*	rs1965707	*SFTPB*	rs1130866	*SFTPC*	rs1124		0.00000	0.4 (0.3-0.6)
38#	*SFTPA1*	rs4253527	*SFTPA2*	rs1965708	*SFTPB*	rs1130866		0.03626	0.6 (0.4-0.9)
39*	*SFTPA1*	rs4253527	*SFTPD*	rs721917	*SFTPB*	rs1130866		0.01405	1.8 (1.3-2.6)
40#	*SFTPA1*	rs4253527	*SFTPD*	rs2243639	*SFTPC*	rs4715		0.04905	0.6 (0.4-0.9)
41*	*SFTPA1*	rs4253527	*SFTPB*	rs1130866	*SFTPC*	rs1124		0.03323	1.7 (1.2-2.4)
42#	*SFTPA2*	rs1965708	*SFTPD*	rs2243639	*SFTPC*	rs4715		0.00095	0.5 (0.3-0.7)
43#	*SFTPA2*	rs1965708	*SFTPD*	rs2243639	*SFTPC*	rs1124		0.00015	0.4 (0.3-0.6)
44#	*SFTPA2*	rs1965708	*SFTPB*	rs1130866	*SFTPC*	rs1124		0.00001	0.4 (0.3-0.6)
45#	*SFTPA2*	rs1965708	*SFTPC*	rs4715	*SFTPC*	rs1124		0.03506	0.6 (0.4-0.9)
46#	*SFTPD*	rs721917	*SFTPC*	rs4715	*SFTPC*	rs1124		0.03626	0.6 (0.5-0.9)
47#	*SFTPD*	rs2243639	*SFTPC*	rs4715	*SFTPC*	rs1124		0.01402	0.6 (0.4-0.8)

[a] shows intragenic interactions
[b] Interactions among the SNPs of hydrophilic SP SNPs
Sign “*” and “#” show interactions that increased and decreased PDAD risk, respectively.
FDR – False discovery rate
